# Reply to ‘Evidence for simple volcanic rifting not complex subduction initiation in the Laxmi Basin’

**DOI:** 10.1038/s41467-020-16570-5

**Published:** 2020-06-01

**Authors:** Dhananjai K. Pandey, Anju Pandey, Scott A. Whattam

**Affiliations:** 1 National Centre for Polar & Ocean Research, Ministry of Earth Sciences, Vasco da Gama, Goa, 403804 India; 2 H-V-3, NCPOR Campus, Vasco da Gama, Goa, 403804 India; 3 Department of Geosciences, King Fahd University of Petroleum & Minerals, Dhahran, 31261 Saudi Arabia

**Keywords:** Geochemistry, Geophysics, Tectonics

## Abstract

Recently, Pandey et al. proposed relict subduction initiation occurred along a passive margin in the northwest Indian Ocean, however, Clift et al. questioned their evidence for subduction initiation, suggesting that simpler rifting-related processes could more simply explain the available data. Here, Pandey et al. reply to Clift et al.’s comment, and argue that geochemical and isotope data for Laxmi basin lavas distinctly imply relict subduction initiation.

**Replying to** Clift et al. *Nature Communications* 10.1038/s41467-020-16569-y (2020)

Pandey et al.^1^ proposed relict subduction initiation in the Laxmi Basin (LB) as a viable alternative to numerous competing tectonic models for the evolution of western Indian margin. We thank Clift et al.^2^ for their interest in our study. They contend that basalts from volcanic rifted margins may also exhibit analogous geochemical signatures. However, we do not agree with their views for several reasons.

Clift et al.^2^ argue that enrichments in certain elements such as Th, U, Pb and Sr, typical of subduction magmatism, may also imply possible crustal assimilation. However, they^2^ overlooked the extent of such enrichments. In fact, the scale of Th enrichments for both lava types differ almost by an order of magnitude^2^. Low average Th/Yb ratios for LB (~0.2) are linked to a shallower source, e.g., mid-ocean ridge basalt (MORB), similar to subduction related ophiolites^3^. In contrast, rifted margin basalts exhibit much higher Th/Yb ratios (>1) pointing at much deeper sources e.g., ocean island basalts. Similarly, TiO_2_/Yb vs. Nb/Yb systematics^2^ of the LB and IBM forearc both overlap significantly with notably low TiO_2_ contents—distinct from volcanic margin basalts.

Clift et al.^2^ further state that Nd/Sr isotopes may indicate continental input instead of subduction initiation (SI)^1^ during its petrogenesis. Their contention is, however, inconsistent when examined with existing isotopic data. Average εNd values^4^ of lower and upper continental crust are −10 and −20 respectively while global subducting sediments (GLOSS) is restricted below −5. In contrast, LB lavas, having εNd values between +6 to +8, are very close to Depleted Mantle. Moreover, average Ba concentrations of continental crust, LB lava and Muslim Bagh ophiolites are ~259, 140 and 110 ppm^4^ respectively^4^. Therefore, isotopic, total trace and rare earth elements (REE) characteristics of LB lava are akin to SI, instead of volcanic margins. Although Clift et al.^2^ did not include isotopic data for comparison, we suspect that the ^87^Sr/^86^Sr values from rifted volcanic margins would be significantly higher than the modest LB lava values (7037–0.7044), which are similar to Neotethyan ophiolites and forearc lavas^1^.

Highly mobile elements (e.g., Rb, Sr, Ba, U) are concentrated into aqueous slab-derived fluids, whereas Th and other light rare earth elements (LREE) are partitioned into sediment-derived melts^5–7^. Enrichment in Ba and to minor extent Th again points towards SI. Similarly, Ba/Th vs. Th variations^1^ show evidence of shallow, fluid-derived enrichments^8^. Using unaltered MORB dataset of Jenner and O’Neill^9^, one can clearly see enrichment of Ba in LB lava relative to MORB. Often Sr and Ba additions can result from seawater hydrothermal alteration processes, however LB lavas show no discernible correlation between Sr, Ba and Loss on ignition (*R*^2^ = 0.01 for both). In fact, samples with the lowest loss on ignition values, exhibit the highest Ba. This implies that the high Ba relative to MORB of the LB lavas, similar to the Muslim Bagh ophiolite, is a primary feature of LB lavas and the result of shallow slab-derived fluid input and not seawater alteration.

The most important distinction between LB and volcanic margin basalts come from REE signatures, which Clift et al. have ignored^2^. LB lavas exhibit highly depleted LREEs and low total REEs in contrast to volcanic margin lava (see Fig. 1). Crustal inputs at volcanic margins typically result in significantly elevated concentrations of large ion lithophile elements and LREEs, which are absent in LB (Fig. 1). A plot of chondrite normalized [La/Yb]_*N*_ ratios vs. SiO_2_ (Fig. 2) also highlights differences with significantly higher [La/Yb]_*N*_ values for North Atlantic Igneous Province lavas (~0.6 to as high as 30) in comparison to LB lavas (~0.6–1.4). The lack of LREE enrichments in LB lava (similar to forearc basalts of the IBM with [La/Yb]_*N*_ less than 1.4) is therefore noteworthy. Furthermore, boninitic-like lava from LB exhibit characteristic U-shaped REE profiles which are primarily attributed to proto-forearc and forearc environments^10^.

**Fig. 1:**
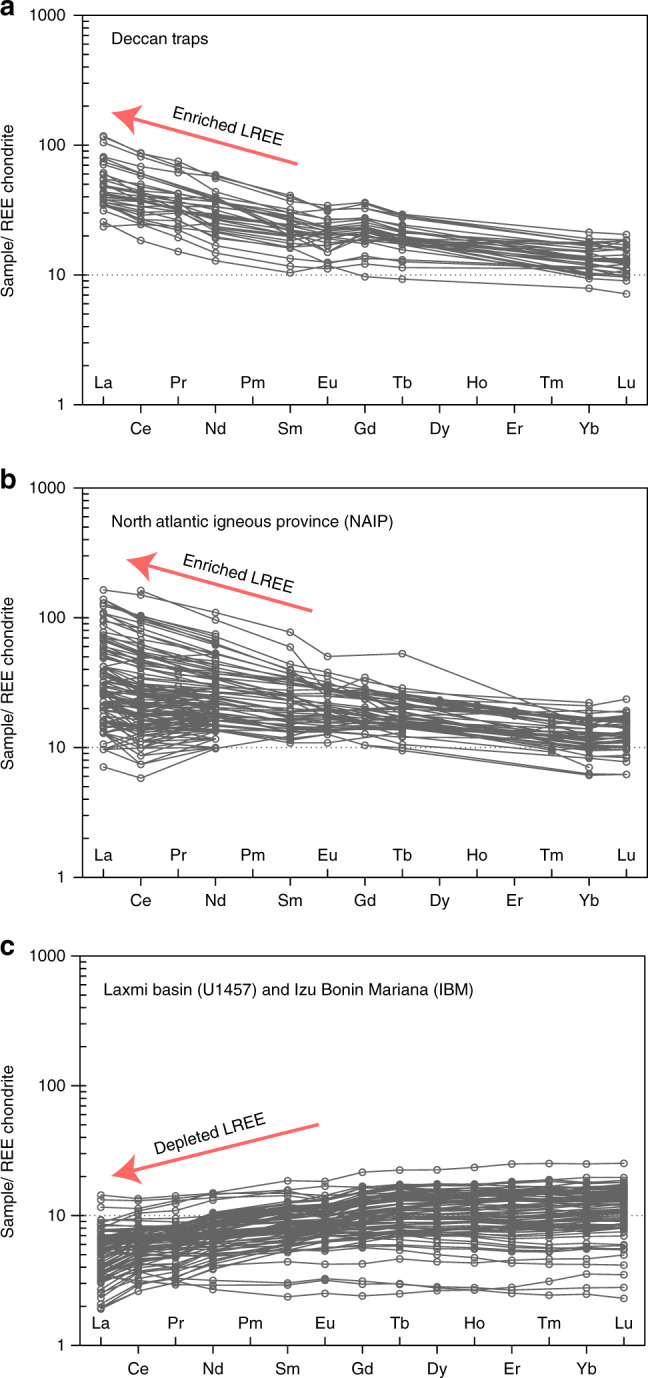
REE variations of different lava types. Chondrite–normalized rare earth element variations of different lava types. **a**–**c** The above diagram highlights characteristic differences among lavas from different tectonic regimes as labelled. Arrows indicate general enrichment/depletion trends. (See Pandey et al.^1^ and Clift et al.^2^ for various data sources).

**Fig. 2:**
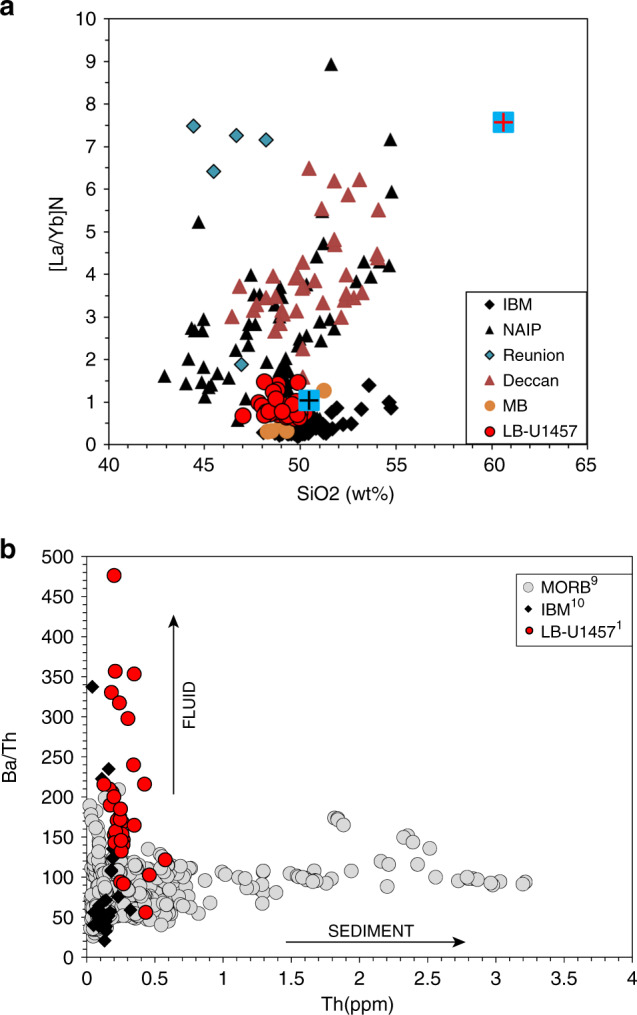
Role of hydrous fluids in LB lava. Characteristic geochemical diagrams. **a** Plot of chondrite normalized [La/Yb]_*N*_ ratios vs. SiO_2_ for different lava types. The black and red ‘+’ signs embossed in blue squares represent mid-ocean ridge basalt (MORB)^9^ and bulk continental crust^4^ ratios respectively. **b** Ba/Th vs. Th abundances for LB lava to evaluate role of hydrous fluids versus sediments during subduction process. High Ba/Th values with low Th abundances are widely attributed to preferential mobilization of large ion lithophile elements (LILEs e.g., Ba) through hydrous fluids components (vertical array) as opposed to the sediment flux (horizontal array) from the downgoing slab. IBM: Izu-Bonin-Mariana; NAIP: North Atlantic igneous province; MB: Muslim Bagh. (See Pandey et al.^1^ and Clift et al.^2^ for various data sources).

Further, TiO_2_ is among the least mobile elements and compared to other trace elements, solubility of TiO_2_ is very low in common mantle or subduction zone fluids. TiO_2_ concentrations may depend on the rate of crystallization of Fe-Ti phases. With respect to high LREE, Th/Yb and enrichment of Ti, we argue that the LREE (and HREE) enrichments relative to MREE seen in U-shaped REE profiles is typical of boninitic-like lavas and is not related to crustal contamination or alteration.

Prior studies from this region envisaged numerous intra-oceanic weak zones at ~70 Ma (see Pandey et al.^1^). Rapid northward drift of India coupled with frequent rotations since the late Cretaceous period is well established. After its spectacular journey from the southern latitudes, India presently resides in the northern hemisphere. Accordingly, considerable amount of missing/unaccounted for crust in the NW Indian Ocean inferred by kinematic modelling implies synchronous compressional tectonics in this region (see Pandey et al.^1^).

Clift et al.^2^’s comments are largely drawn from a particular school of thoughts based on indirect geophysical models proposed well before drilling in LB in 2015. Using same regional seismic profiles^11–13^ different groups interpret Laxmi ridge and basin variably either as continental/oceanic crust. Equivocal crustal models remained inconclusive about whether this margin is a magma rich or magma poor type. Likewise, sporadic intra-basement magmatic reflections on seismic profiles are interpreted as seaward dipping reflectors (SDR)/axial anomalies/extinct spreading centres/volcanic flows/ intrusives etc^1, 11–13^. No precise knowledge is available regarding basin-wide extent of SDRs in the LB, in contrast to what is reported by Clift et al^2^. Therefore, a sweeping portrayal^2^ of SDRs in LB and surrounding regions appears to be conjectural.

Identification of a proto-trench would require detailed morphological and crustal imaging of the region, in time and space, which was beyond the scope of Pandey et al^1^. Clift et al.^2^ further link formation of the Laxmi ridge to that of the Saurashtra Volcanic Province. Due to inconclusive interpretations and lack of any samples/rocks/data from both regions, we prefer not to speculate about its precise affinity. However, geochemical signatures of LB lava are categorically different from that of Deccan volcanics^14^.

Two-dimensional flexural modeling primarily dealt with post-rift evolution of the LB (awaiting geochemical results from Site U1457) and confirmed significant residual bathymetry in LB and surrounding regions (~2-3 km at ~61 Ma). This means that any prior magmatism must have occurred under considerably deep-water settings, in consonance with shipboard observations about rapid quenching of the LB basalts after their emplacement. Regional crustal uplift however, would depend upon several factors including the onset, extent and duration of the impingement of a potential thermal regime.

Finally, we would like to conclude by pointing out that our study^1^ reports direct data from LB through crustal sampling for the first time. Geochemical and isotopic imprints of LB lava distinctly imply relict subduction initiation. Therefore, new findings based on direct observations cannot be undermined merely to support specific geophysical models. Indeed, additional regional basement sampling, radiometric dating and revised plate kinematic modelling would provide important insights about the complexity of the western Indian margin.

## Data Availability

No new data are reported in this manuscript. See Pandey et al.^1^ and Clift et al.^2^ for data sources related to Fig. 1 and 2.
